# Integrated and high-throughput method to collect, store, recover, and manage microbial isolates in mini-arrays

**DOI:** 10.1128/spectrum.02637-24

**Published:** 2025-03-04

**Authors:** Moon H. Nahm

**Affiliations:** 1 Division of Pulmonary Allergy, and Critical Care Medicine, Department of Medicine, University of Alabama at Birmingham, Birmingham, Alabama, USA; Emory University School of Medicine, Atlanta, Georgia, USA

**Keywords:** cryopreservation, bacteria, *Streptococcus pneumoniae*, microbe array culture

## Abstract

Clinical studies of vaccines generally require collections of microbial isolates obtained from various body sites over multiple years. Further, large microbe collections are needed for research due to increasing appreciation for the phenotypic and genotypic diversity among a single microbial species. However, large collections are not generally available due to method limitations. We show a new way to create, recover, and manage microbe collections in 96- or 384-well plates using 50% glycerol at −20°C. Fifty percent glycerol remains liquid at −20°C and permits only the chosen isolates to be reliably sampled without first thawing all other isolates in the plate. Consequently, the glycerol sampling allows integration of microbe collection, labeling, recovery, and storage steps. Creating a microbe collection as an array in microplates reduces physical storage space by 6- or 23-fold with 96-well or 384-well plates, respectively. The array permits direct analysis of the collection with high-throughput assay systems. Further, we show that *Streptococcus pneumoniae* could be stored for 11 years as a microplate array. Standardized microbe arrays created in microplates with the new method could be easily distributed for studies of microbial structures, genetic diversity, antibiotics, and microbe–host interactions. This integrated method suggests how automated microbe management systems can be created.

**IMPORTANCE:**

Epidemiologic and microbiology studies require large microbial collections, and the use of microplates could facilitate the creation and management of these collections. However, recovering individual isolates from microplates is manual and tedious. In this study, we demonstrate a simple method for recovering a selected individual isolate from a microplate at −20°C using 50% glycerol. Additionally, we found that *Streptococcus pneumoniae* could be revived for more than 10 years in microplates. This new method of recovering microbes from frozen microplates could greatly streamline many large-scale epidemiologic studies, particularly those related to pneumococcal vaccine studies. This new method may ultimately automate the collection, management, and storage of microbial isolates.

## INTRODUCTION

Even a single species of a microbe is quite diverse. For example, *Escherichia coli* and *Streptococcus pneumoniae* (the pneumococcus) have more than 100 capsule types each (1–5) and have pangenomes exceeding 1,000 representative strains (6) (Personal communication with H. Tettelin). In addition, the isolates of both bacterial species display large diversity in virulence or metabolic properties. Thus, a large collection of isolates of a single species is of fundamental importance to microbiology research ranging from scientific studies of pathogen virulence to industrial developments of new foods (7).

Demonstration of the effectiveness of a vaccine requires monitoring epidemiology of a microbe for a long time. For instance, the use of pneumococcal vaccines requires long-term epidemiologic monitoring of isolates obtained from patients suffering from invasive pneumococcal infections or otitis media or isolates carried among adults or children. Furthermore, serotype shifts may occur with vaccine usage and can be observed if the epidemiologic monitoring is performed for a long time. Consequently, there is a great need for collecting and managing a large number of microbial isolates efficiently. Many nations have created national organizations to perform large epidemiologic studies.

Conventional approaches to collect and manage microbial isolates use cryovials, which contain one isolate per vial, are individually labeled, and are individually stored. Microbial collections often have numerous (>10^4^) cryovials, and retrieving a select isolate from the large cryovial bank is laborious. Thus, creating, storing, and managing a large microbe collection is challenging.

Large collections could be managed efficiently if the isolates could be collected and stored in a physical array, like a microplate. However, microplates have not been used for isolate collections because a selected isolate could not be recovered without thawing all other isolates in the plate, and the re-culture following the unnecessary thaw would introduce genetic drifts among the isolates (8). To sample only the selected isolates in a plate without thawing the whole plate, we have developed the “glycerol sampling” method and now show how this innovative sampling method integrates the collection, storage, and analysis of microbial isolates. Further, we show that the microbe array in microplates permits reliable strain identification, storage space reduction, and long-term storage, as well as automatic handling of the isolates and the use of high-throughput assays.

## MATERIALS AND METHODS

### Culture media and solutions

Todd Hewitt (TH) broth, Luria–Bertani (LB) broth, and tryptic soy broth (TSB) were prepared using appropriate commercial formulations following manufacturer’s instructions. Todd Hewitt broth with yeast extract (THY) was prepared by adding 5 g of yeast extract to 1 L of TH broth. Yeast extract peptone dextrose (YPD) medium was prepared by adding 10 g of yeast extract, 20 g of peptone, and 20 g of dextrose into 1 L of water. All ingredients were dissolved well before the media were sterilized with a bottle top filter (MilliporeSigma SCGPT05RE from Thermo Fisher Scientific Inc.) and stored at room temperature.

To prepare agar plates with various media, LB agar and Tryptic soy blood agar were prepared using appropriate commercial formulations following manufacturer’s instructions. THY agar and YPD agar were made by adding 15 g of agar into 1 L of THY or YPD broth. All ingredients were mixed well and autoclaved for sterilization before being aliquoted into square Petri dishes.

Eighty percent glycerol was prepared by adding 80 g of glycerol into water to create a final volume of 100 mL and then autoclaving the solution. Then, 50% and 30% glycerol solutions were prepared by diluting 80% glycerol with an appropriate amount of relevant culture broth, and 50% glycerol was kept at 4°C.

### Microbial strains

Various *S. pneumoniae* strains, which include all the pneumococcal capsule types (9), and the *Saccharomyces cerevisiae* strain (MNY133) (10) were Nahm laboratory strains. SSISP1 strain was from Statens Serum Institut in Copenhagen, Denmark. *E. coli* strain ATCC12014 (11), *S. aureus* strains ATCC49525 and ATCC 6538, *Shigella sonnei* strain ATCC 9290, and the *Shigella dysenteriae* strain ATCC 9750 were obtained from ATCC (Manassas, Virginia). *S. agalactiae* strains COH1 and M781 were from Dr. Carol Baker in Houston, Texas. *E. coli* strain ER2357 used for phage display was from New England Biolabs (Ipswich, Mass). *S. pyogenes* strain GAS1 was a laboratory strain from Dr. Susan Hollingshead, and GAS3 was a clinical isolate (pharyngitis) from Dr. Bill Benjamin at UAB. The strains are summarized in Table S1.

### Salt–ice tray

**Fig 1:**
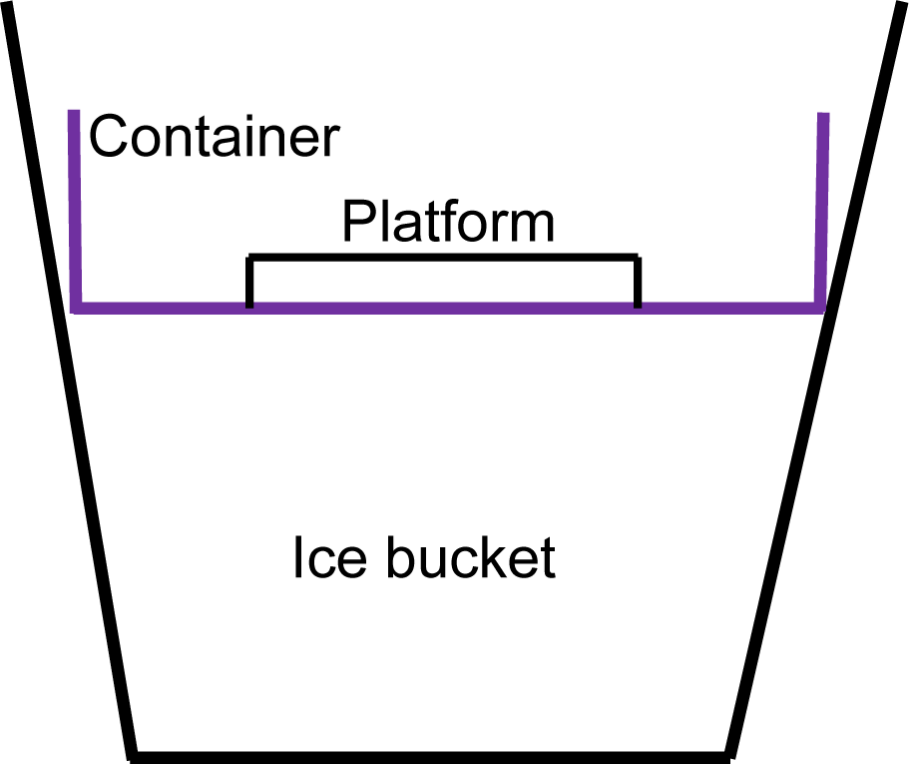
Diagram of the salt–ice tray. The plastic container dimensions are 16 × 16 × 6.5 cm. The ice bucket dimensions are 24 cm at the top and 14 cm at the bottom with a 19 cm height. Ice chips are filled up to the container level, and the salt–ice mixture is placed in the container.

A small plastic platform was placed at the bottom of a plastic container (16 × 16 × 6.5 cm) (Fig. 1). The platform was a part of an ELISA kit (Component 80-0060) from Enzo Life Sciences (Long Island NY) and was used to hold the microbe array plate in place while the ice melts. The plastic container was filled with 100 g of sodium chloride (S271-10 from Thermo Fisher Scientific Inc.) and 300 g of ice chips. Then, the plastic container was placed on the ice in an ice bucket (Bel-Art Magic Touch 2 Ice Bucket from Thermo Fisher Scientific Inc.) filled with ice chips.

### Preparation of microbe array in microplates

Microbe suspensions for a microbe array were prepared by inoculating minitubes (Axygen MTS1112CRS from Thermo Fisher Scientific Inc.) filled with 500 µL of culture medium. The inoculated minitubes were placed in the accompanying holding rack (Axygen MTS1112CRS from Thermo Fisher Scientific Inc.) and incubated in a CO_2_ incubator for a desired time (~5 h – overnight). Incubation time and culture medium were selected according to the microbial species. Then, 500 µL of 30% glycerol was added to each tube. After mixing, 200 µL of a microbe suspension with 15% glycerol was added to each well of a sterile 96-well plate (Falcon 353072 from Thermo Fisher Scientific Inc.). (At this step, one could prepare multiple replicate microbe array plates with one minitube.) The 96-well microplates for storing a microbe array were labeled with barcodes that were generated with a barcode printer (Labelwriter 400 DYMO). The plates were sealed with PCR plate sealer (Nunc AB0580 from Thermo Fisher Scientific Inc.) and placed in a −80°C freezer for long-term storage. When 384-well plates (Nunc 242757 from Thermo Fisher Scientific Inc.) were used, and 80 µL of the microbe suspension with 15% glycerol was added to each well.

### Recover microbes from the stored microbe array

Sterile 50% glycerol in THY or other relevant medium was prepared and kept at 4°C. A selected micro-array plate was moved from −80 °C to −20 °C. After ~1–1.5 h at −20°C, the plate was placed on the platform in the salt–ice tray kept in a biological hood. After removing the sealer, 25 µL of 50% glycerol was placed on the top, and the plate was covered with a sterile plate lid. After 5 min of incubation, ~25 µL of the liquid was removed from the top and mixed with 500 µL of THY in minitubes. After the sampling, the micro-array plate was resealed with the PCR sealer and placed in the −80°C for future use.

The number of microbes in the sampled solution was determined as described (12). Briefly, the sampled solution was properly diluted, and 10 µL was placed on THY agar in a square Petri dish (Greiner Bio-One 688102 from Thermo Fisher Scientific Inc.). When the plate was dry, it was overlaid with an overlay agar with 25 µg/mL of triphenyl tetrazolium chloride (MilliporeSigma T8877) (12) and cultured overnight in the 5% CO_2_ incubator at 37°C. The next day, the agar plates with bacterial colonies were photographed with a digital camera, and the picture was processed with NICE—a free colony counting software (13)—to obtain the number of colony-forming units (CFUs) in each spot.

### Multiplexed immunoassay for pneumococcal serotypes

*Streptococcus pneumoniae* strains recovered from a 12-year-old microbe array were serotyped with a multiplexed serotyping assay that was developed in-house (9). The array contains 94 pneumococcal strains representing 94 different serotypes. The assay is a 26-fold multiplexed inhibition-type assay designed to detect 26 pneumococcal vaccine-relevant serotypes. The capsule in the sample inhibits 26 different capsule types coated on 26 color-coded microbeads, which are reacting with 26 different monoclonal antibodies recognizing different capsule types. The amount of monoclonal antibody bound to the beads was measured with a flow cytometer and plotted against the samples.

## RESULTS

### The primary cultures in micro-wells can be repeatedly sampled

We hypothesized that each well of microtiter plates could be sampled with 50% glycerol at −20°C because the primary culture, which typically contains 15% glycerol, should remain frozen, but 50% glycerol should be liquid at that temperature. To test the “glycerol sampling” method, we prepared 96-well plates with two different pneumococcal isolates. Briefly, serotype 1 and 3 pneumococcal isolates were cultured in THY broth to OD_600_ of 0.8, which corresponded to ~4 × 10^8^ and ~4 × 10^7^ CFU/m respectively. To investigate the impact of various bacterial densities on the performance of the sampling method, we prepared pneumococcal cultures diluted 10- and 100-fold. After mixing the three (neat, 1:9, and 1:99) different bacterial suspensions with an equal volume of 30% glycerol, 200 µL of each mixture was placed in six wells of a 96-well microtiter plate, and the plates were stored in a −80°C freezer.

**Fig 2:**
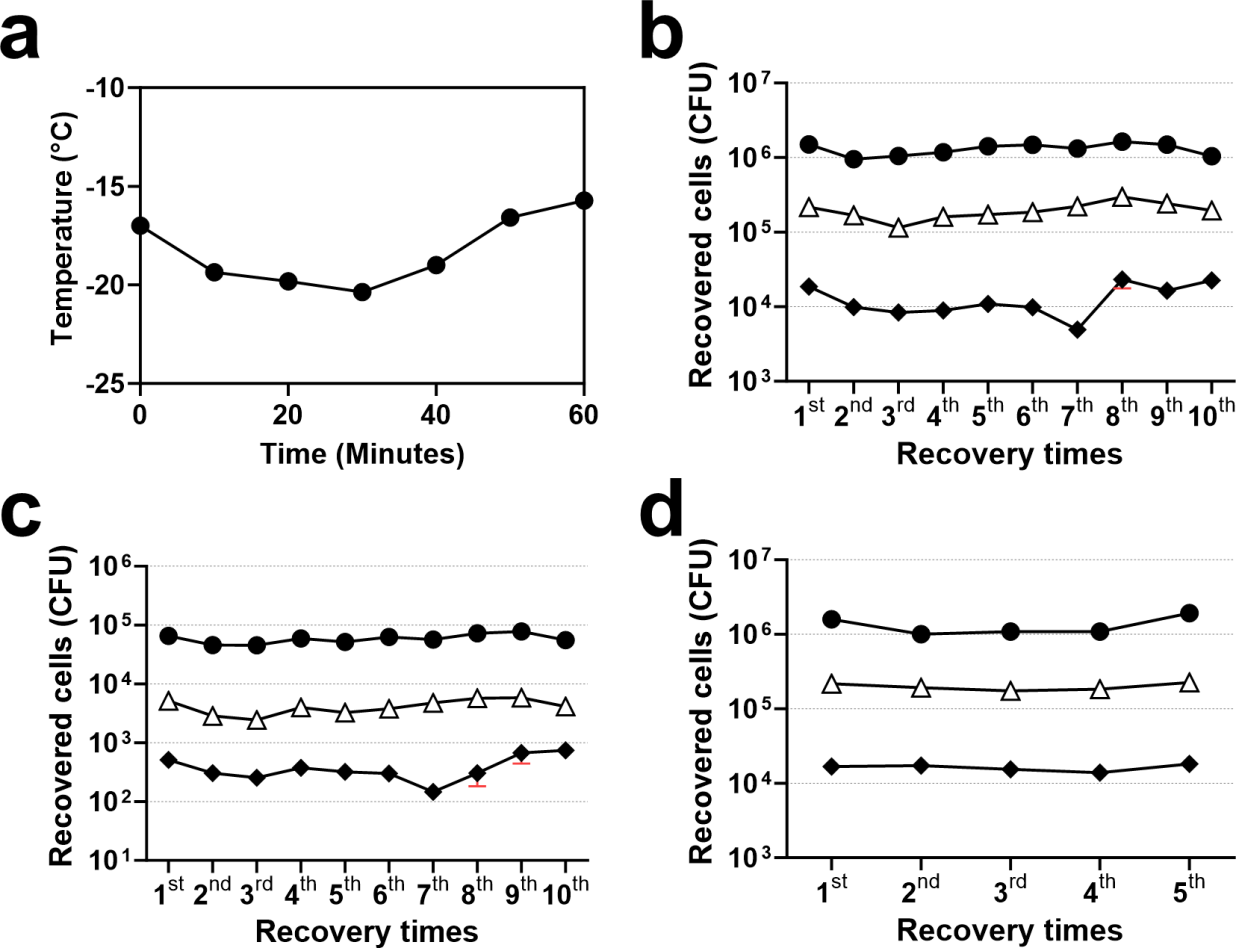
Thermal properties of the salt–ice tray and repeat sampling from microwells. Panel (a) shows the temperature of the salt–ice tray over time. Panels (b, c, and d) show recovery of two different strains of pneumococci from the primary cultures in 96-well plates (panels b and d) or in 386-well plate (panel d) over multiple times of recovery. The primary cultures have *S. pneumoniae* serotype 1 strain in panels (b) and (d) and serotype 3 strain in panel (c). Primary cultures with different bacterial densities were created by no dilution (solid circle), 10-fold dilution (open triangle), and 100-fold dilution (solid diamond). Each data point shows the mean and standard error (red bars) of the six wells. Since the standard error was small, the symbols hide the red bars for most data points except for several data points.

To perform the glycerol sampling in a tissue culture hood, we created a small “salt–ice tray” using a mixture of sodium chloride and crushed ice at 1:3 by weight as described in the method section. The salt–ice tray maintained a microtiter plate at −15–20 °C for about 1 h (Fig. 2a), and the glycerol sampling of a microplate required less than 30 min. To sample isolates, the plate was removed from −80°C and placed in a −20°C freezer for an hour before being placed on a salt–ice tray. Then, 25 μL of 50% glycerol at 4°C was placed at the top of the frozen primary cultures. After 5 min, the 25 µL was removed from the top, and the number of pneumococci in the 25 µL was determined. Abundant numbers of bacteria were recovered in all cases (Fig. 1c and 2b), and the number of recovered bacteria was largely proportional to the bacterial density in the primary culture. For instance, the first recovery yielded about 10^6^ CFUs from the undiluted serotype one culture and about 1.9 × 10^4^ CFUs from the 1:99 diluted culture. After the first sampling, we then investigated if the primary cultures can be repeatedly sampled. The rate of recovery was maintained throughout the 10 samplings (Fig. 1c and 2b), and the volume of the frozen primary culture remaining in the micro-wells was not appreciably reduced after 10 samplings.

To investigate the glycerol-based sampling more quantitatively, we determined the numbers of bacteria frozen and recovered using two strains of *S. pneumoniae* (Table S2). To estimate the recovery rate, it was assumed that the sampled amount of the frozen bacteria is equal to the amount of glycerol added to each well. While this assumption is likely a high estimate, a significant fraction of bacteria could be recovered (6%−18%), and the recovery rates were independent of the number of input bacteria as noted above.

### Various microbes can be sampled after prolonged storage in 96-well plates

To test if microbes can be recovered after a prolonged cryopreservation in micro-wells, we created a 96-well plate with multiple strains of various microbial species: 11 strains of *S. pneumoniae*; two strains each of *Streptococcus pyogenes*, *Streptococcus agalactiae*, *Staphylococcus aureus*, *E. coli,* and *Shigella dysenteriae*; and one strain of *Saccharomyces cerevisiae* (Table 1). Bacterial densities of each culture were not monitored to reflect practical situations. When we recovered the strains after 6 months of storage, all bacterial strains yielded more than 2.5 × 10^4^ CFUs and 16 (out of 22) yielded more than 2.5 × 10^5^ CFUs. Reflecting the low cell density in the original culture, 375 CFUs could be recovered from the yeast strain. Nevertheless, many microbes can be stored for a prolonged period in micro-wells.

**TABLE 1:** Recovery of microbes after 6 months^*a*^

Species	Isolates tested	96-well plate		384-well plate	
		Pre-dilution	CFU observed	Pre-dilution	CFU observed
*S. pneumoniae*	11	100X	>250	100X	>250
*S. pyogenes*	2	100X	>250	100X	>250
*S. agalactiae*	2	100X	>250	100X	>250
*S. aureus*	2	100X	>250	100X	>250
*E. coli*	2	100X	>250	100X	>250
*S. sonnei*	1	100X	>250	100X	>250
*S. dysenteriae*	1	100X	>250	100X	>250
*S. cerevisiae*	1	5X	75	10X	>250

^*a*^ This study used strains ATCC12014 and ER2357 for *E. coli*, strains ATCC49525 and ATCC 6538 for *S. aureus*, strains COH1 and M781 for *S. agalactiae*, strains GAS1 and GAS3 for *S. pyogenes*, strain ATCC9290 for *S. sonnei*, and ATCC9750 for *S. dysenteriae*, and strain MNY133 for *S. cerevisiae*. The strains are further described in Table S1.

### The new sampling method can reliably sample isolates in 96-well plates

An important advantage of this integrated method is its ability to handle many samples. To exploit this advantage, however, the sampling method should be able to reliably sample numerous isolates. Thus, we investigated the sampling reliability by sampling 960 wells (i.e., ten 96-well microplates) of a pneumococcal isolate (Table 2). When each primary culture well was sampled as described above, more than 2.5 × 10^5^ CFUs were recovered from 959 wells. The one well that provided 1.7 × 10^4^ CFU provided more than 2.5 × 10^5^ CFUs upon repeat sampling, suggesting that the initial low recovery was due to pipetting error. Thus, the glycerol sampling method is reliable enough to handle a very large number of primary cultures.

**TABLE 2:** Reliability of recovering microbes after 1 week

Species^*b*^	Culture medium	From 96-well plates			From 384-well plates		
		Well number	Pre-dilution	CFU observed	Well number	Pre-dilution	CFU observed
*S. pneumoniae*	THY	960^*a*^	1000X	> 250	192	1000X	>250
*S. aureus*	THY	192	100X	> 250	96	100X	>250
	LB	192	1000X	> 250	ND^*c*^	ND	ND
	TSB	192	1000X	> 250	ND	ND	ND
*E. coli*	THY	192	1000X	> 250	96	1000X	>250
	LB	192	1000X	> 250	ND	ND	ND
	TSB	192	1000X	> 250	ND	ND	ND
*S. cerevisiae*	YPD	192^*a*^	5X	> 250	96	5X	>250

^*a*^ One well of *S. pneumoniae* provided 1.7 × 10^4^ CFU in the first sampling but provided more than 2.5 × 10^5^ CFU in the second trial. One well of yeast produced 850 CFU initially but produced more than 1,250 CFU in repeat sampling. The initial recovery failures are probably due to causes other than cryopreservation (e.g., a pipetting error).

^*b*^ The strains used for the study are SSISP1 for *S. pneumoniae*, ATCC49525 for *S. aureus*, ATCC12014 *for E. coli,* and MNY133 for *S. cerevisiae*.

^*c*^ ND means not done.

To show the generality of our sampling method, we investigated sampling reliability with other microbial species including Gram-positive, Gram-negative, and yeast strains grown in various culture media. *S. aureus* and *E. coli* strains were grown to be visibly turbid (OD_600_ => 1) before cryo-preservation, and a *S. cerevisiae* strain was grown for 7 h to an OD = 1.0, which corresponded to 7.6 × 10^6^ CFU/mL. Sampling reproducibility was tested with 192 cultures of each isolate. As shown in Table 2, more than 2.5 × 10^4^ CFUs of bacteria and 1,250 CFU of yeast were recovered from all 192 wells regardless of the culture media. The recovery was even better if we exclude the results with *S. aureus* grown in THY broth. Thus, while the exact cryopreservation condition should be optimized for the species of interest, the glycerol sampling method works reliably for diverse microbial species.

### The glycerol sampling technique works with 384-well microplates

Microtiter plates with 384 wells would be more efficient in storage than 96-well plates. We have therefore investigated the glycerol sampling method with specimens stored in 384-well plates. The sampling was performed exactly as described for 96-well plates but using 20 µL of 50% glycerol. The primary culture used for the 384-well plates had about 10^8^ CFU/mL before dilution. As shown in Fig. 2d, pneumococci could be readily recovered multiple times, and the number of recovered bacteria was comparable to those from 96-well plates. Also, large numbers of bacteria and yeast could be recovered even after 6 months of cryo-preservation (Table 1). Further, the sampling method can recover microbes reliably from many wells containing Gram-positive bacteria, Gram-negative bacteria, or yeast isolates (Table 2). Thus, the 384-well plates are also an acceptable array platform for this method.

### Pneumococci could be recovered from an array after 11 years of cryopreservation

**Fig 3:**
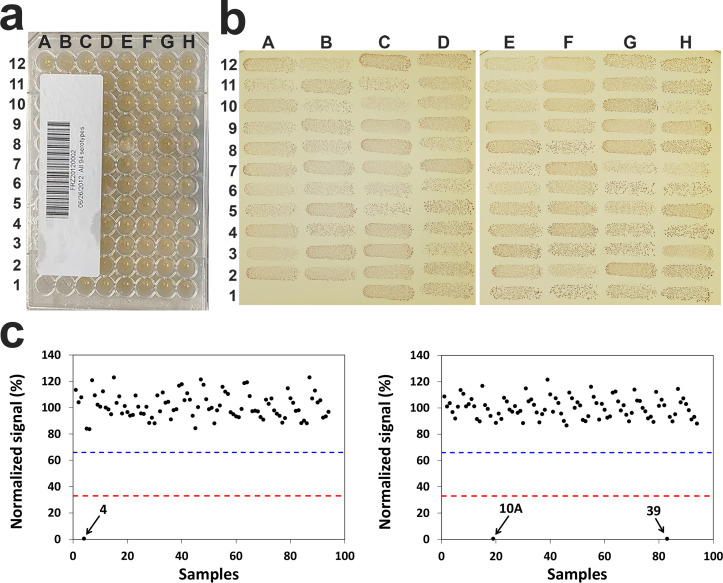
Recovery of *S. pneumoniae* after 11 years of storage at −80°C. Panel (a) shows the 96-well plate containing 94 pneumococcal strains representing 94 different pneumococcal serotypes. The microplate was labeled with a bar code for identification. Panel (b) shows bacterial colonies from 94 wells. Two wells in the bottom left (**A1 and B1**) were negative controls with no bacteria. All 94 wells produced more than 100 CFU after 1,000-fold dilution. Panel (c) shows the results of the 94 wells for serotype 4 (left) and serotype 10A (right) assays. Results from other serotypes were not shown. The red and black dashed lines respectively indicate the 33% and 66% of the maximum average signals (i.e., signal with no inhibition). The serotype 4 assay was inhibited by the one correct sample. The 10A assay is known to react with both serotypes 10A and 39 (9) and showed strong inhibition by the two samples: serotypes 10A and 39.

Pneumococci produce large amounts of polysaccharide capsule that is serotype specific, and the pneumococcal collection can be used as a glyco-array (14). To create a pneumococcal glycobiome, 11 years ago, we prepared a 96-well plate with pneumococcal isolates expressing 94 different pneumococcal serotypes (9) and kept the plate at −80°C (Fig. 3A). To investigate if a microbe array can be recovered after a very long period of storage, we recovered the bacteria from the plate using the glycerol sampling technique. We were able to recover more than 2.5 × 10^4^ CFUs of pneumococci from all the 94 wells (Fig. 3b) and more than 2.5 × 10^5^ CFUs from 90 wells.

To further demonstrate that the microbe array can be used in conjunction with high-throughput analytical methods, we determined the capsule types of the pneumococcal isolates recovered from the plate using a 26-fold multiplexed, microplate-based immunoassay for pneumococcal serotypes we previously developed (9). As shown in Fig. 3c, all 94 samples produced the expected results. For example, serotype 4 reacted with the antibody against serotype 4. Serotypes 10A and 39 reacted with the anti-10A antibody, as described before (9). Taken together, bacterial isolates can be stored in microplates for a very long time, and microplate arrangement of the isolates facilitates their evaluation with high-throughput analyses.

## DISCUSSION

Herein, we describe a novel sampling method of retrieving selected isolates in a 96- or 384-well microplate without thawing the primary cultures. We also show that the method can be used to store diverse microbes as microplate arrays (ranging from bacteria to yeast at various microbial densities) for long periods. In case of *S. pneumoniae*, it could be regrown after storage in microplates for at least 11 years. We also describe how the new method results in a new technology for microbial collection and storage by integrating the collection, storage, and management of microbes. While additional research is needed, the new technology, termed the microbe array culture technology, will have fundamental value for microbiology and infectious disease research.

One immediate benefit of the microbe array culture technology is a ~ 6–23-fold reduction in freezer storage space, addressing both cost and space limitations. Furthermore, each isolate can be identified by its position in bar-coded microplates, which greatly streamlines both sample labeling and retrieval. These benefits increase storage reliability since replica plates can be prepared and stored in multiple sites. Another significant benefit is the genetic preservation of the primary cultures because the primary cultures can be sampled without the thaw and re-culture cycle, which is known to cause genetic drift (8). Previously, the re-culture cycle could be avoided by preparing primary cultures with glass beads (15, 16) or manually scraping the top of the primary cultures (17). These methods are unsuitable for automation.

An exciting future development would be to create an instrument that automates the entire process of microbial isolate collection, preservation, and recovery of selected isolates. The instrument would greatly expand the microbe array culture technology by increasing the size of microbial collections that can be collected and managed. To create the instrument, one should replace our “salt–ice tray” with a metallic device providing a dry surface with a temperature of −20°C, perhaps based on Peltier junction devices. We used the tray due to its simplicity, but its surface is wet and not suitable for automation. The instrument may have the ability to lyophilize the entire microplate array. To do this, one should replace the glycerol with some other volatile compound.

Given the widespread use of microtiter plates in automated high-throughput analytical methods, such as ELISA or flow cytometry, facilitated by barcoded microplates and automated pipettes, microbe arrays simplify the investigation of large microbial collections, which are commonly encountered in large epidemiologic surveys or experiments involving experimentally induced genetic variants. Moreover, the ability to investigate a large number of isolates would alter experimental strategies. For example, it is unclear if a person carries multiple pneumococcal serotypes since previous analytical limitations forced one to obtain one isolate per person. However, the ability to collect pneumococcal isolates as an array and to semi-automatically serotype them could permit a new epidemiologic approach – sampling multiple isolates from a person.

Microbial arrays leave the primary cultures intact and avoid genetic drift, unlike previous cryopreservation methods that required thawing and re-culturing the entire sample. Further, microbe arrays are compact, easily transportable among different laboratories, and expandable with additional microorganisms displaying unique glycan (e.g., capsule or O-antigen) molecules due to the method’s compatibility with a wide variety of microbial species. Therefore, a carefully constructed microplate array could serve as the “reference” panel of microorganisms with unique molecular features. For instance, a pneumococcal array with different polysaccharide capsule types could function as a glycan array that would be valuable for investigating the glycan specificity of various host lectins (14). Reference microbial arrays may include strains expressing different proteins, genes, or antibiotic resistances of a species. The antibiotic resistance array should be useful in searches for new antibiotics. In addition, the arrays could be useful in studying interactions of one microbe species with another or with eukaryotic cells.

## SUPPLEMENTAL MATERIAL

Supplemental material spectrum.02637-24-s0001.pdf Tables S1 and S2.

ASM does not own the copyrights to Supplemental Material that may be linked to, or accessed through, an article. The authors have granted ASM a non-exclusive, world-wide license to publish the Supplemental Material files. Please contact the corresponding author directly for reuse.
