# Supplementary material for: Integrated and high-throughput method to collect, store, recover, and manage microbial isolates in mini-arrays
Source: Microbiol Spectr. 2025 Mar 4;13(4):e02637-24. doi: 10.1128/spectrum.02637-24 (PMC11960078; doi:10.1128/spectrum.02637-24)
Supplement: Supplemental material — Tables S1 and S2. [file spectrum.02637-24-s0001.pdf]

Supplementary material for:

Integrated and high-throughput method to collect, store, recover, and manage microbial isolates in mini-arrays

Moon H. Nahm

Division of Pulmonary, Allergy, and Critical care, Department of Medicine, University of Alabama at Birmingham

Address correspondence to Moon H. Nahm (mnahm@uabmc.edu)

Supplementary Table S1: List of microbe strains used in the study

Species	Strain name	Reference or source or description
S. pyogenes	GAS1	from Dr. Susan Hollingshead at UAB
S. pyogenes	GAS3	from Dr. Bill Benjamin at UAB.
S. agalactiae	COH1	from Dr. Carol Baker in Houston Texas
S. agalactiae	M781	from Dr. Carol Baker in Houston Texas
S. aureus	ATCC49525	from ATCC
S. aureus	ATCC 6538	from ATCC
E. coli	ATCC12014	from ATCC
E. coli	ER2357	from New England Biolabs (Ipswich, Mass).
S. cerevisiae	MNY133	Nahm Laboratory strain
S. sonnei	ATCC 9290 (MNY45)	from ATCC
S. dysenteriae	ATCC 9750 (MNY46)	from ATCC
S. pneumoniae	MNZ755	Nahm Laboratory strain
S. pneumoniae	MNZ773	Nahm Laboratory strain
S. pneumoniae	MNZ870	Nahm Laboratory strain
S. pneumoniae	MNZ825	Nahm Laboratory strain
S. pneumoniae	CDC3609-06	from US CDC
S. pneumoniae	CDC3032-06	from US CDC
S. pneumoniae	SSISP1	from Statens Serum Institut, Denmark
S. pneumoniae	SSISP2	from Statens Serum Institut, Denmark
S. pneumoniae	SSISP3	from Statens Serum Institut, Denmark
S. pneumoniae	SSISP4	from Statens Serum Institut, Denmark
S. pneumoniae	SSISP5	from Statens Serum Institut, Denmark

Supplementary Table S2: Study of bacteria recovery with 50% glycerol.

Recovery of SSISP1

Dilution	Pre-freeze concentration (cfu/ml)	cfu per microwell (200 µl)	Expected* Recovery (cfu/50µl)	Observed recovery (cfu/50µl)	Recovery (%)
None	2.31E+08	4.62E+07	1.16E+07	1.50E+06	13.00%
10 fold	2.31E+07	4.62E+06	1.16E+06	2.16+05	18.70%
100 fold	2.31E+06	4.62E+05	1.16E+05	1.85+04	16.10%
1000 fold	2.31E+05	4.62E+04	1.16E+04	1.93+03	16.70%

Recovery of SSISP3

Dilution	Pre-freeze concentration (cfu/ml)	cfu per microwell (200 µl)	Expected* Recovery (cfu/50µl)	Observed recovery (cfu/50µl)	Recovery (%)
None	1.59E+07	3.17E+06	7.93E+05	6.56E+04	8.30%
10 fold	1.59E+06	3.17E+05	7.93E+04	5.15E+03	6.50%
100 fold	1.59E+05	3.17E+04	7.93E+03	5.13E+02	6.50%
1000 fold	1.59E+04	3.17E+03	7.93E+02	8.30E+02	10.50%

* For the recovery calculation, it was assumed that 50 µl of glycerol would melt 50 µl of the frozen culture medium containing bacteria.

** The experiment was performed using 50 µl of 50% glycerol per well and a microtiter plate filled with 200 µl of frozen bacteria per well. The calculation of the expected recovery rate was performed with the assumption that 50 µl was molten and recovered with 50 µl of glycerol. With this assumption, we obtained about 6-18% of the expected number of bacteria. Our assumption is obviously a high estimate as we noted that the volume of the frozen well did not visibly change after multiple sampling.
